# Case Report: High-grade endometrial adenocarcinoma with somatically derived yolk sac tumor differentiation

**DOI:** 10.3389/fonc.2026.1904556

**Published:** 2026-09-03

**Authors:** Juan Hao, Xiaomeng Wang, Min Cai, Shuxu Tian, Huangbei Song, Xuan Liu

**Affiliations:** 1 Department of Gynecology, Qingdao Women and Children’s Hospital, Qingdao University, Qingdao, China; 2 Department of Obstetrics, The Affiliated Hospital of Qingdao University, Qingdao, China; 3 Department of Pathology, Qingdao Women and Children’s Hospital, Qingdao University, Qingdao, China

**Keywords:** AFP, case reports, endometrial adenocarcinoma, somatically derived yolk sac tumor, yolk sac tumor-like differentiation

## Abstract

**Background:**

Yolk sac tumors (YSTs) arising from somatic tumors of the female genital tract present with variable clinical manifestations depending on the organ of origin. Their diagnosis is highly challenging due to their rarity and the limited number of well-documented cases, and their prognosis remains unclear. The coexistence of high-grade endometrial adenocarcinoma with somatically derived yolk sac tumor differentiation (SDYST) is extremely rare, and evidence-based treatment strategies remain limited.

**Case highlights:**

A 44-year-old premenopausal woman presented with abnormal uterine bleeding predominantly characterized by menorrhagia for approximately two months. Following surgical staging, pathological examination revealed high-grade endometrial adenocarcinoma with SDYST. Immunohistochemistry (IHC) showed positivity for SALL4 and Glypican-3 (GPC3), with focal expression of alpha-fetoprotein (AFP). Serum AFP levels were markedly elevated.

**Follow-up and outcomes:**

The patient recovered well postoperatively. After multidisciplinary evaluation, she received BEP-based chemotherapy, and serum AFP levels were dynamically monitored. Serum AFP decreased markedly during treatment, and no definite evidence of disease progression was observed during short-term follow-up.

**Lessons:**

This case provides a practical clinical lesson: markedly elevated serum AFP in an aggressive endometrial tumor should prompt consideration of SDYST, particularly when yolk sac tumor-like morphology and positive immunohistochemical staining for AFP, SALL4, and GPC3 are present. Early recognition of this phenotype may facilitate appropriate immunohistochemical workup, AFP-based disease monitoring, and timely multidisciplinary treatment.

## 1 Introduction

Yolk sac tumor (YST) is a malignant germ cell tumor (GCT) that morphologically resembles the fetal yolk sac. Rutgers et al. first reported the association between YST differentiation and ovarian epithelial carcinoma, specifically ovarian endometrioid carcinoma (1). YST most commonly arises in the ovaries of young women, with a median age at diagnosis of 17–22 years, and typically occurs as either a pure or mixed germ cell tumor (1, 2). However, YSTs may also arise at other sites, including the uterine corpus, cervix, vulva, and vagina (3). Malignant tumors with YST differentiation are rare and have distinct clinicopathological significance in women over 40 years of age (4). In this age group, YST differentiation may occur in apparently pure YSTs, mixed germ cell tumors, or tumors associated with non-germ cell malignant components, most commonly Müllerian-derived carcinomas (5, 6). YSTs have also been reported in association with postmenopausal mucinous cystadenofibroma (7), mucinous carcinoma (8), low-grade serous tumors (9), borderline clear cell adenofibroma, and large cell neuroendocrine carcinoma (5).

The diagnosis and management of endometrial cancer increasingly rely on the integrated assessment of histopathological features and molecular classification, including mismatch repair (MMR) status, p53 expression pattern, and, when available, POLE mutation status, to identify tumor subtypes with different biological behaviors and prognostic risks. In high-grade endometrial carcinoma, markedly elevated serum alpha-fetoprotein (AFP) may provide an important diagnostic clue to somatically derived yolk sac tumor differentiation (SDYST), and immunohistochemical markers such as SALL4, Glypican-3 (GPC3), and AFP may assist in the differential diagnosis. Although SDYST is extremely rare, evidence remains limited regarding its aggressive behavior, risk of distant metastasis, and optimal postoperative treatment. Here, we report a case of a 44-year-old premenopausal woman who presented with abnormal uterine bleeding and was diagnosed after surgery with stage IVB high-grade endometrial adenocarcinoma with SDYST. After comprehensive staging surgery and adjuvant chemotherapy with the BEP regimen, the patient’s serum AFP level decreased markedly, and no definite evidence of disease progression was observed during follow-up approximately 10 months after surgery.

## 2 Case report

### 2.1 Case presentation

The patient was a 44-year-old premenopausal Han Chinese woman with a history of cesarean section. She was 155 cm in height and weighed 54–55 kg, with a body mass index of approximately 22.9 kg/m². She denied any family history of malignancy. She had previously tested positive for human papillomavirus type 52 (HPV52) and had no history of genetic testing. Her menstrual cycles had previously been regular, with a cycle length of 26 days and menstruation lasting 3–5 days.

In May 2025, the patient noticed increased menstrual bleeding, with the menstrual volume approximately twice her usual amount and the menstrual period prolonged to 8–10 days. She did not report intermenstrual or postcoital bleeding at that time. Subsequently, she developed persistent vaginal spotting and episodes of heavy vaginal bleeding with blood clots, with the bleeding volume estimated to be 4–5 times her usual amount. She also reported low-grade fever and lower abdominal distension.

**Figure 1:**
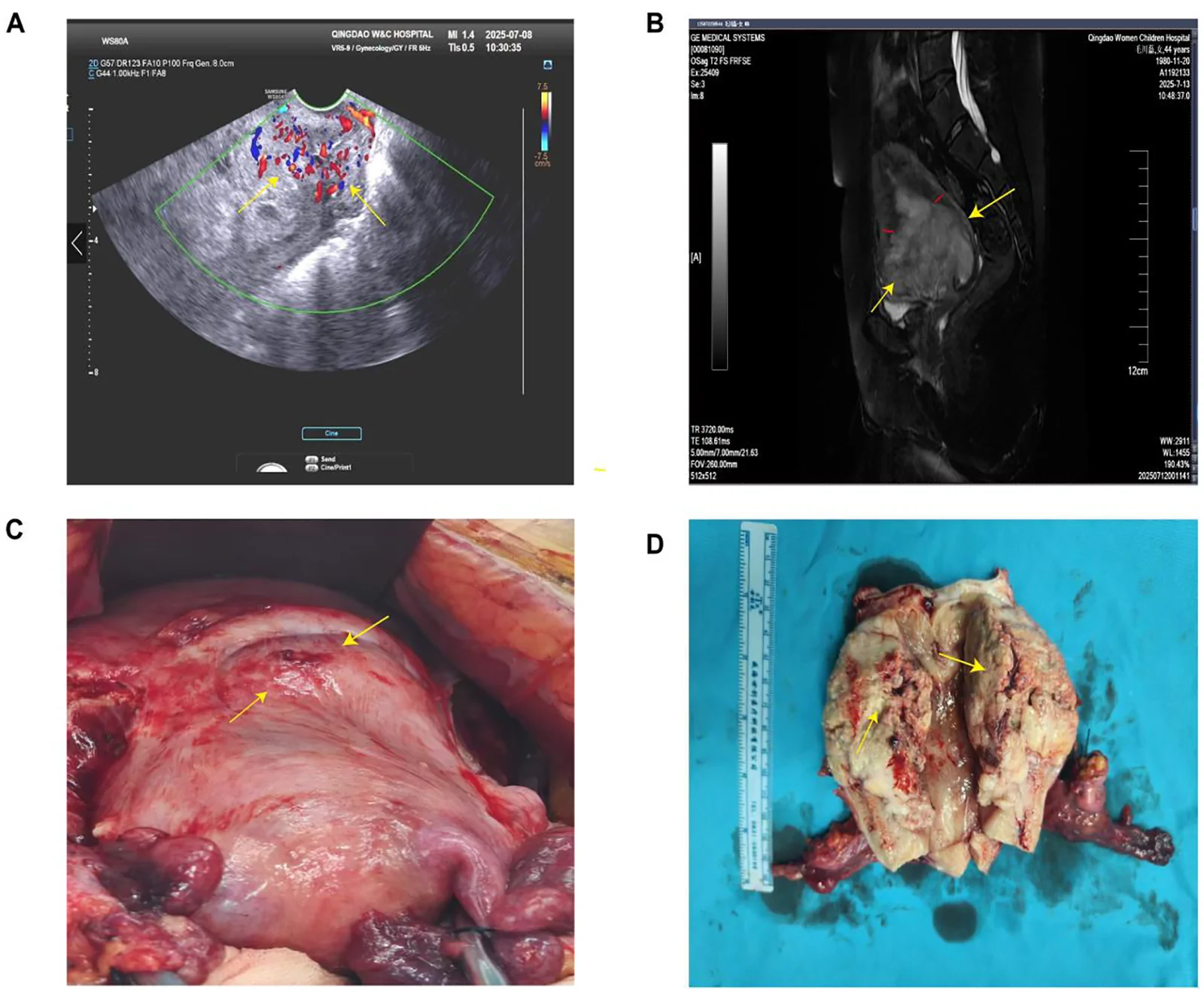
Imaging, intraoperative, and gross findings. **(A)** Transvaginal color Doppler ultrasonography showed a heterogeneous intrauterine mass with abundant intratumoral vascularity. **(B)** Pelvic MRI showed a large intrauterine mass with myometrial involvement and suspected serosal extension. **(C)** Intraoperative view showed an enlarged uterus with focal serosal congestion and bulging, suggesting marked expansile tumor growth and possible serosal involvement. **(D)** Gross specimen revealed a grey-white, friable intrauterine tumor with extensive hemorrhagic and necrotic areas, consistent with rapid tumor growth and aggressive biological behavior.

Transvaginal ultrasonography revealed a heterogeneous mass within the uterine cavity, measuring approximately 81×57×54 mm. Color Doppler imaging showed abundant intratumoral vascularity, with a resistance index of 0.53 (**Figure 1A**). Pelvic MRI showed a large intrauterine mass with myometrial involvement and suspected serosal extension (**Figure 1B**). Laboratory tests showed leukocytosis and elevated inflammatory markers, with a white blood cell count of 17.01×10^9^/L and a C-reactive protein level of 60.74 mg/L. Serum AFP was markedly elevated at 7879.00 ng/mL. The levels of CA125, CA19-9, and HE4 were 27.10 U/mL, 37.94 U/mL, and 80.20 pmol/L, respectively. Gastrointes- tinal endoscopy revealed no evidence of a digestive tract tumor.

### 2.2 Diagnosis and treatment

Hysteroscopic resection of the intrauterine lesion was performed on July 15, 2025. Intraoperatively, the uterine cavity was found to be distorted and filled with yellow-white friable tissue with prominent neovascularization. The lesion extended to the internal cervical os. Colposcopic biopsy revealed LSIL/CIN1 at the 4 o’clock position of the cervix and focal HSIL/CIN3 at the 8 o’clock position. Endocervical curettage revealed a malignant tumor, and yolk sac tumor was considered as a possible diagnosis.

On July 21, 2025, the patient underwent abdominal modified radical hysterectomy, bilateral salpingo-oophorectomy, pelvic and para-aortic lymph node dissection, omentectomy, and resection of visible abdominal lesions. Intraoperatively, the uterus was enlarged to the size of a 2-month pregnancy, with focal serosal congestion and bulging suggestive of possible serosal involvement (**Figure 1C**). This serosal bulging was considered to reflect marked expansile tumor growth and possible transmural extension, which was consistent with the subsequent pathological finding of serosal penetration. Nodular lesions were observed in the greater omentum and transverse mesocolon.

**Figure 2:**
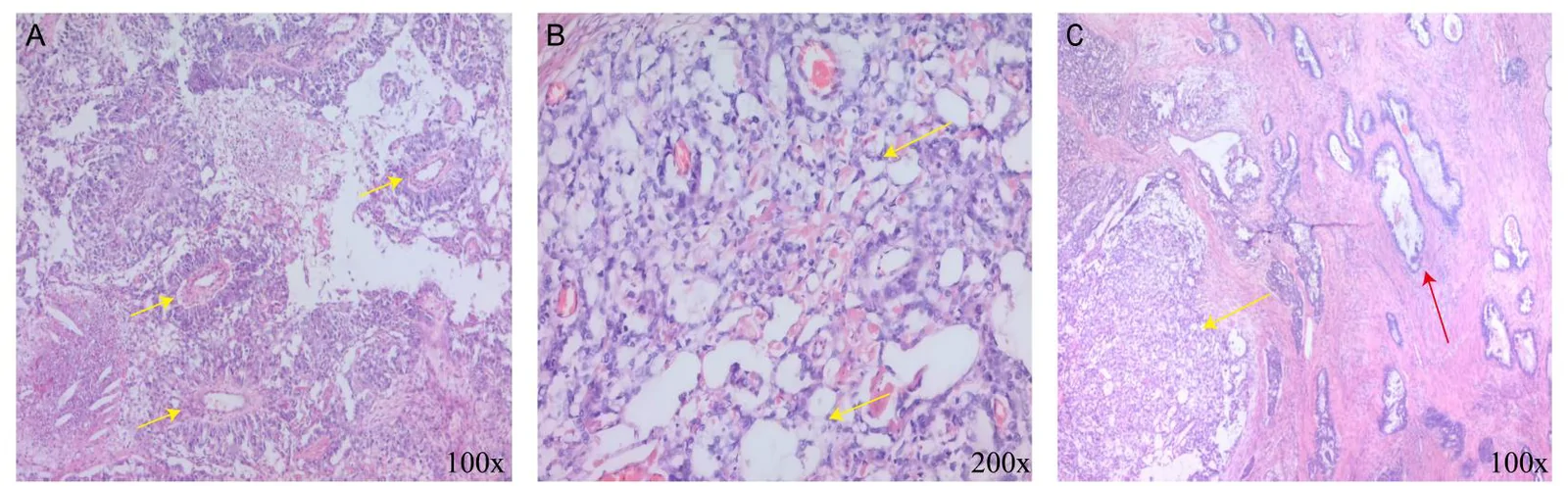
Histopathological findings of the tumor on H&E staining. **(A)** Schiller-Duval body-like structures were observed within the tumor, as indicated by yellow arrows. Original magnification: ×100. **(B)** Higher-power view showed yolk sac tumor-like differentiation with reticular/microcystic patterns, indicated by yellow arrows. Original magnification: ×200. **(C)** Coexisting yolk sac tumor-like component and adenocarcinoma component were identified. The yellow arrow indicates the yolk sac tumor-like component, and the red arrow indicates the adenocarcinoma component. Original magnification: ×100.

Gross examination of the resected uterus showed a grey-white, friable intrauterine tumor with hemorrhagic and necrotic areas (**Figure 1D**). The extensive hemorrhage and necrosis suggested rapid tumor proliferation and insufficient tumor perfusion, supporting the clinically aggressive nature of the lesion. Pathological examination revealed high-grade endometrial adenocarcinoma with SDYST. On H&E-stained sections, the tumor contained two morphologically distinct but closely related components. The yolk sac tumor-like component accounted for approximately 90% of the tumor and showed Schiller-Duval body-like structures and reticular/microcystic patterns (**Figures 2A, B**). The high-grade endometrial adenocarcinoma component accounted for approximately 10% of the tumor. The two components were intimately admixed, with coexisting yolk sac tumor-like and adenocarcinoma components identified in the same tumor area (**Figure 2C**). Although no definite histological transition was identified, the intimate admixture of the two components and the coexistence of a Müllerian adenocarcinoma component supported SDYST rather than a collision tumor. These findings supported the diagnosis of high-grade endometrial adenocarcinoma with SDYST. The tumor measured 9×7.5×4 cm. It penetrated the uterine serosa, invaded the surrounding adipose tissue, and involved the inner one-third of the cervical stroma. Lymphovascular invasion was also identified. Metastatic tumor deposits were found in the transverse mesocolon and greater omentum. Lymph node assessment revealed metastasis in one of nine left pelvic lymph nodes and isolated tumor cells in one of nine right pelvic lymph nodes. No metastasis was identified in the para-aortic or presacral lymph nodes.

**Figure 3:**
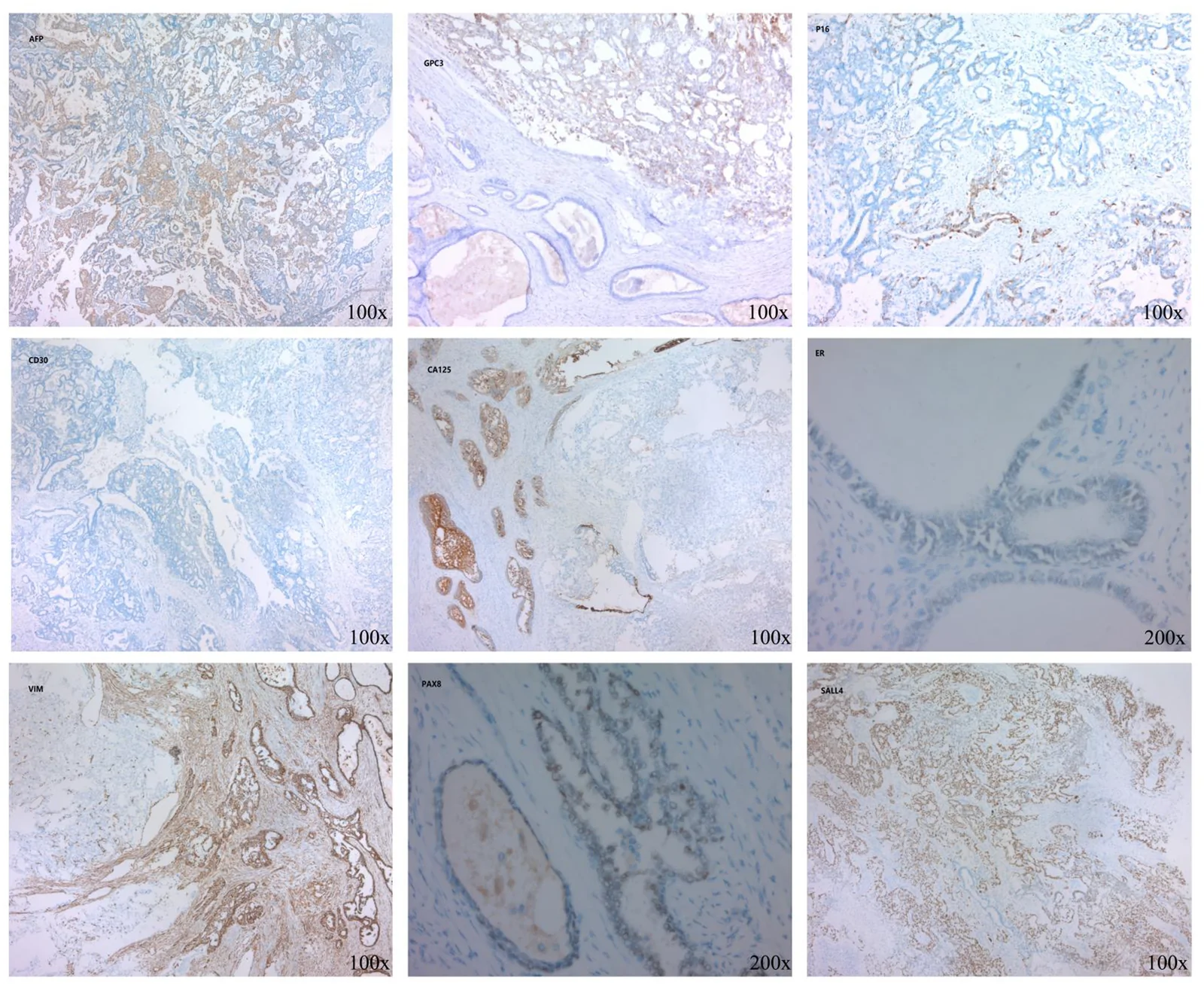
Immunohistochemical profile of the tumor. Representative immunohistochemical staining showed focal AFP expression, diffuse glypican-3 expression, and positive SALL4 staining in the yolk sac tumor-like component. PAX8 and ER showed focal nuclear positivity, supporting a Müllerian epithelial origin. CA125 was positive in selected epithelial tumor areas, vimentin was partially positive, p16 showed a patchy expression pattern, and CD30 was negative.

Immunohistochemical staining was performed using the primary antibodies listed in **Supplementary Table 1**. Immunohistochemical staining showed that the yolk sac tumor-like component was positive for SALL4 and glypican-3, with focal AFP expression (**Figure 3**). CKpan was diffusely positive in the tumor cells, supporting epithelial differentiation. Focal expression of CK7, PAX8, ER, and PR was observed, supporting a Müllerian epithelial origin. p53 showed a wild-type expression pattern, and the Ki-67 proliferation index was approximately 60% in hotspot areas. The mismatch repair proteins MLH1, PMS2, MSH2, and MSH6 were retained. Although AFP *in situ* hybridization was not performed, the diagnosis was supported by the integration of typical yolk sac tumor-like morphology, markedly elevated serum AFP, and positive immunohistochemical staining for AFP, SALL4, and GPC3. In the appropriate clinicopathological context, these combined findings were considered sufficient to establish yolk sac tumor differentiation within high-grade endometrial adenocarcinoma.

Based on these findings, the patient was diagnosed with stage IVB high-grade endometrial adenocarcinoma with SDYST according to the 2023 FIGO staging system. Adjuvant chemotherapy was initiated on August 13, 2025, using a 21-day BEP regimen, which consisted of bleomycin 30 mg on days 1 and 4, etoposide 150 mg on days 1–5, and cisplatin 100 mg on day 1. To date, the patient has completed five cycles of chemotherapy.

### 2.3 Follow-up and outcomes

The patient recovered well after surgery without obvious postoperative complications. Postoperative serum AFP before chemotherapy was 1436.50 ng/mL and then decreased to 464.10 ng/mL, 30.5 ng/mL, 8.3 ng/mL, and finally 6.9 ng/mL during chemotherapy. CA19–9 levels remained mildly elevated, ranging from 32.72 to 40.55 U/mL. Other tumor markers showed no obvious abnormalities.

During chemotherapy, complete blood counts and biochemical profiles were monitored before each chemotherapy cycle. The lowest recorded hemoglobin level, absolute neutrophil count, and platelet count were 82 g/L, 0.25 ×10^9^/L, and 193 ×10^9^/L, respectively. Renal function remained stable, with serum creatinine ranging from 47.89 to 60.39 μmol/L and creatinine clearance ranging from 87.01 to 109.72 mL/min. No clinically significant hepatic dysfunction was observed, with peak ALT and AST levels of 21.03 and 22.54 U/L, respectively. According to CTCAE v5.0 criteria, the patient developed transient grade 4 neutropenia but did not experience febrile neutropenia, grade≥3 anemia or thrombocytopenia, severe gastrointestinal toxicity, nephrotoxicity, neurotoxicity, or treatment-related hospitalization. Because the regimen included bleomycin, respiratory symptoms and pulmonary toxicity were closely monitored during treatment. Pulmonary function tests showed no clinically significant decline in FVC or FEV1. No cough, dyspnea, decreased oxygen saturation, or imaging evidence suggestive of bleomycin-related pulmonary toxicity was observed.

The last follow-up was performed on May 22, 2026, corresponding to approximately 10 months after surgery and 9 months after initiation of adjuvant chemotherapy. At the last follow-up, the patient remained alive without definite clinical evidence of disease progression.

At the last follow-up, serum AFP remained within the normal range, supporting a sustained biochemical response. However, because the follow-up duration remains limited, long-term recurrence risk, survival outcomes, and durability of treatment response require further observation. The timeline of the patient’s diagnosis, intervention, and follow-up process is shown in **Table 1**.

**Table 1:** Clinical timeline of diagnosis, treatment, and follow-up.

Time point	Clinical phase/event	Key actions & interventions	Findings & outcomes
May 2025	Symptom onset	Patient developed abnormal uterine bleeding and menorrhagia.	Symptoms persisted for approximately two months, with low-grade fever and lower abdominal distension.
Preoperative evaluation	Imaging and laboratory assessment	Transvaginal ultrasound, pelvic MRI, tumor markers, and gastrointestinal endoscopy were performed.	A large hypervascular intrauterine mass was detected. Serum AFP was markedly elevated at 7879.00 ng/mL. No digestive tract tumor was found.
July 15, 2025	Hysteroscopic resection	Hysteroscopic resection, colposcopic biopsy, and endocervical curettage were performed.	Malignant tumor was identified, and yolk sac tumor was considered.
July 21, 2025	Staging surgery	Abdominal modified radical hysterectomy, BSO, lymph node dissection, omentectomy, and resection of visible lesions were performed.	Nodular lesions were found in the greater omentum and transverse mesocolon.
Postoperative pathology	Final diagnosis	Histopathology and immunohistochemistry were performed.	High-grade endometrial adenocarcinoma with SDYST was confirmed. Yolk sac tumor-like component accounted for approximately 90%.
Postoperative staging	FIGO staging	Metastatic deposits and lymph nodes were assessed.	Stage IVB disease was diagnosed according to the 2023 FIGO staging system.
August 13, 2025	Chemotherapy initiation	BEP chemotherapy was started.	Postoperative AFP before chemotherapy was 1436.50 ng/mL.
During chemotherapy	Treatment monitoring	Serial AFP monitoring and toxicity assessment were performed.	AFP decreased to 464.10, 30.5, 8.3, and 6.9 ng/mL. Transient grade 4 neutropenia occurred, but no febrile neutropenia, treatment-related hospitalization, or clinically significant pulmonary, renal, hepatic, or neurologic toxicity was observed.
May 22, 2026	Last follow-up	Clinical evaluation, serum AFP monitoring, and imaging assessment were performed.	Alive without definite clinical or radiological evidence of disease progression, approximately 10 months after surgery and 9 months after initiation of adjuvant chemotherapy.

AFP, alpha-fetoprotein; MRI, magnetic resonance imaging; BSO, bilateral salpingo-oophorectomy; BEP, bleomycin, etoposide, and cisplatin; FIGO, International Federation of Gynecology and Obstetrics; IHC, immunohistochemistry; SDYST, somatically derived yolk sac tumor differentiation.

## 3 Discussion

SDYST is exceedingly rare in the female reproductive tract and has nonspecific clinical manifestations, which may lead to confusion with common endometrial carcinoma, intrauterine infection, or tumor necrosis. The present case was diagnosed as high-grade endometrial adenocarcinoma with SDYST, consistent with a somatically derived yolk sac tumor (10, 11). The patient initially presented with abnormal uterine bleeding. Imaging revealed a large solid intrauterine mass with abundant vascularity, extending into the cervical canal. She also had a low-grade fever, leukocytosis, and an elevated C-reactive protein level, suggesting an inflammatory response possibly related to tumor necrosis or secondary infection. Notably, serum AFP was markedly elevated early in the disease course, reaching 7879 ng/mL. Postoperative pathology confirmed high-grade endometrial adenocarcinoma with SDYST, supporting the association between AFP elevation and the tumor’s differentiation pattern. These findings also indicate that AFP may serve as a useful marker for treatment response assessment and follow-up monitoring (12).

Pathological examination showed that the tumor was predominantly composed of yolk sac tumor-like components, with Schiller-Duval body-like structures, glandular yolk sac tumor patterns, and reticular/microcystic areas. Some areas morphologically resembled clear cell carcinoma or poorly differentiated endometrial carcinoma; however, the corresponding immunophenotype did not support these diagnoses. Focal areas at the tumor margin showed endometrioid adenocarcinoma-like glandular morphology, with PAX8 positivity and weak ER expression, suggesting a possible origin from endometrial epithelial tumor components. Immunohistochemistry showed positivity for SALL4 and GPC3 in the yolk sac tumor-like areas, with focal AFP expression. Focal CK7, PAX8, ER, and PR expression further supported a Müllerian epithelial origin, suggesting that the yolk sac tumor-like component was somatically derived from endometrial carcinoma rather than representing a primary germ cell tumor. The main differential diagnoses included hepatoid adenocarcinoma, AFP-producing endometrial carcinoma without definite yolk sac tumor differentiation, clear cell carcinoma, and metastatic gastrointestinal or hepatocellular carcinoma. Hepatoid adenocarcinoma was less likely because typical hepatoid morphology was absent, whereas yolk sac tumor-like structures were present. Metastatic gastrointestinal or hepatocellular carcinoma was also unlikely given the negative gastrointestinal endoscopy, uterine-centered tumor location, and coexistence of a Müllerian adenocarcinoma component. Based on the combined evidence of typical yolk sac tumor-like morphology, markedly elevated serum AFP, positivity for AFP, SALL4, and GPC3, and the coexistence of a Müllerian epithelial carcinoma component, the final diagnosis was high-grade endometrial adenocarcinoma with SDYST. Although AFP *in situ* hybridization was not available, the integrated morphological, serological, and immunohistochemical findings were sufficient to support this diagnosis.

Previous reports have described mutant-pattern p53 expression in some SDYST cases (4), however, p53 showed a wild-type expression pattern in the present case, suggesting possible molecular heterogeneity in this tumor type. This finding may indicate that SDYST does not represent a single molecularly uniform entity and may arise through different molecular pathways. Nevertheless, the clinical significance of p53 wild-type expression in SDYST remains unclear because of the limited number of reported cases. In this case, the p53 wild-type pattern did not directly change the treatment strategy, which was mainly determined by the advanced stage and the presence of yolk sac tumor-like differentiation. Further cases with TP53 sequencing, POLE mutation analysis, and long-term follow-up are needed to clarify whether p53 status has prognostic or therapeutic relevance in SDYST. The absence of comprehensive molecular profiling also limited the interpretation of tumor biology in the present case. Although p53 showed a wild-type immunohistochemical pattern and retained expression of MLH1, PMS2, MSH2, and MSH6 suggested mismatch repair proficiency by immunohistochemistry, PCR- or sequencing-based MSI testing was not performed. Therefore, the tumor could not be definitively assigned to a contemporary molecular subgroup of endometrial carcinoma. This limitation may affect prognostic assessment and adjuvant treatment decision-making. For example, POLE-mutated tumors may have a more favorable prognosis, whereas p53-abnormal tumors are generally associated with more aggressive behavior. Although retained MMR protein expression makes mismatch repair deficiency less likely in the present case, independent MSI testing and broader molecular profiling would provide a more complete basis for assessing potential immunotherapy eligibility and hereditary cancer predisposition, including Lynch syndrome. Based on currently available evidence, SDYST should be interpreted cautiously as a yolk sac tumor-like differentiation phenotype arising in association with somatic endometrial carcinoma, rather than as a fully established independent molecular entity. Accumulation of additional molecularly characterized cases is needed to clarify whether SDYST represents a distinct molecular category or a phenotypic variant within existing endometrial carcinoma subtypes.

To better contextualize the present case, we reviewed previously reported uterine or endometrial tumors with SDYST. The relevant cases are summarized in **Supplementary Table 2**. Because the present tumor arose in the endometrium, ovarian epithelial tumors with YST differentiation were not included in the main summary table, although they provide important historical context for the concept of SDYST.

Given the limited number of reported SDYST cases, no standard treatment strategy has been established. Management is therefore usually individualized, taking into account tumor stage, histological composition, serum AFP level, and the patient’s general condition. Carboplatin plus paclitaxel is commonly used for advanced or high-risk endometrial carcinoma and was considered in the treatment discussion. However, in the present case, the tumor showed predominant yolk sac tumor-like differentiation, markedly elevated AFP, and metastatic disease. These findings suggested that the germ cell tumor-like component might represent a biologically aggressive and clinically monitorable component of the tumor. Because BEP is widely used for YSTs and other malignant germ cell tumors, the regimen was selected after multidisciplinary discussion to target the germ cell-like component. Thus, BEP was not regarded as a standard regimen for SDYST, but as an individualized treatment choice in the absence of established evidence for this rare tumor type.

During chemotherapy, the patient was regularly monitored by complete blood count, liver and renal function tests, serum tumor markers, and clinical evaluation for pulmonary, renal, neurologic, and hematologic toxicities. Serum AFP showed a continuous decline, suggesting its value as a marker for chemotherapy response. In contrast, conventional endometrial cancer-related markers, including CA125, CA19-9, and HE4, were normal or only mildly elevated and did not show a comparable dynamic trend. These findings suggest that AFP may be more informative than conventional endometrial cancer markers for treatment response assessment and follow-up surveillance in tumors with yolk sac tumor differentiation. Although the BEP regimen may provide therapeutic benefit, it is associated with potential toxicities, including bleomycin-related pulmonary toxicity, cisplatin-related nephrotoxicity and neurotoxicity, and etoposide-related myelosuppression. In the present case, transient grade 4 neutropenia occurred according to CTCAE v5.0, but the patient did not develop febrile neutropenia, grade≥3 anemia or thrombocytopenia, treatment-related hospitalization, or clinically significant pulmonary, renal, hepatic, or neurologic toxicity. Hematologic, renal, hepatic, neurologic, and pulmonary toxicities were monitored throughout treatment, and the overall adverse events remained clinically manageable. These safety findings suggest short-term tolerability of BEP in this patient, although longer follow-up and more cases are required before any general conclusion can be made. Treatment and follow-up strategies should therefore be adjusted dynamically according to the patient’s tolerance, organ function, tumor burden, and multidisciplinary assessment. The patient completed five cycles of chemotherapy and remained alive without definite clinical or radiological evidence of disease progression at the last follow-up on May 22, 2026.

## 4 Conclusion

This case highlights the diagnostic and therapeutic challenges of high-grade endometrial adenocarcinoma with SDYST. In adult women, particularly women over 40 years of age, presenting with abnormal uterine bleeding, a hypervascular intrauterine mass, and markedly elevated serum AFP, SDYST should be considered. Clinically, the combination of disproportionate serum AFP elevation, aggressive endometrial tumor behavior, yolk sac tumor-like morphology, and positive immunohistochemical staining for AFP, SALL4, and GPC3 should prompt active consideration of SDYST. Even when the tumor appears to arise from the endometrium, it should not be regarded simply as a primary germ cell tumor; somatic differentiation or dedifferentiation from an epithelial malignancy should also be taken into account. Adequate tissue sampling, careful histopathological assessment, and immunohistochemical analysis are therefore essential for an accurate diagnosis. Although no standardized treatment or follow-up strategy has been established, multidisciplinary management and close surveillance with serial AFP monitoring remain reasonable approaches, with treatment benefit and toxicity carefully balanced.

## 5 Patient perspective

The patient reported significant concern about the rarity of the disease, treatment intensity, and prognosis after being diagnosed with advanced high-grade endometrial adenocarcinoma with SDYST. After the physicians explained the pathological diagnosis, the need for surgery, and the subsequent chemotherapy plan, she accepted the comprehensive treatment strategy developed by the multidisciplinary team. During treatment, the continued decline in serum AFP increased her confidence and helped her better understand the importance of follow-up monitoring. She agreed to share her experience to support earlier recognition and clinical management of similar rare tumors.

## 6 Limitations

This single-case report cannot support general conclusions regarding the clinical behavior, optimal treatment, or prognosis of SDYST. The follow-up period remains limited, so long-term recurrence risk and survival outcomes cannot yet be assessed. In addition, although histopathological and immunohistochemical analyses were performed, AFP *in situ* hybridization was not performed, as this assay was not part of routine diagnostic testing at our institution. GPC3 expression was assessed semi-quantitatively by immunohistochemistry rather than by digital quantitative analysis. Comprehensive molecular testing, including POLE, TP53, or broader genomic profiling, was also not conducted. Further cases with molecular data, standardized pathological assessment, and long-term follow-up are needed to guide diagnosis, treatment, and surveillance.

## Data Availability

The original contributions presented in the study are included in the article/**Supplementary Material**. Further inquiries can be directed to the corresponding author.
