# Supplementary material for: Case Report: High-grade endometrial adenocarcinoma with somatically derived yolk sac tumor differentiation
Source: Front Oncol. 2026 Sep 3;16:1904556. doi: 10.3389/fonc.2026.1904556 (PMC13581843; doi:10.3389/fonc.2026.1904556)
Supplement: Supplementary file 1 [file Table1.docx]

| Supplementary Table 1. Primary Antibodies Used for Immunohistochemical Analysis | | | | | | |
| --- | --- | --- | --- | --- | --- | --- |
| Antibody | Clone | Manufacturer | Catalog No. | Ready-to-use | Antigen Retrieval Buffer / pH | Staining Platform |
| AFP | OTI5D2, mouse monoclonal | ZSGB-BIO | ZM-0009 | Yes | EDTA, pH 8.0 | BenchMark ULTRA |
| CD30 | UMAB256, mouse monoclonal | ZSGB-BIO | ZM-0043 | Yes | EDTA, pH 9.0 | BenchMark ULTRA |
| CA125 | OV185:1, mouse monoclonal | ZSGB-BIO | ZM-0019 | Yes | EDTA, pH 9.0 | BenchMark ULTRA |
| ER | EP1, rabbit monoclonal | ZSGB-BIO | ZA-0102 | Yes | EDTA, pH 8.0 | BenchMark ULTRA |
| p16 | 1C1, mouse monoclonal | ZSGB-BIO | ZM-0205 | Yes | EDTA, pH 9.0 | BenchMark ULTRA |
| PAX8 | EP298, rabbit monoclonal | ZSGB-BIO | ZA-0666 | Yes | EDTA, pH 9.0 | BenchMark ULTRA |
| SALL4 | OTI4D7, mouse monoclonal | ZSGB-BIO | ZM-0393 | Yes | EDTA, pH 8.0 | BenchMark ULTRA |
| Vimentin | OTIR5D6, rabbit monoclonal | ZSGB-BIO | ZA-0511 | Yes | EDTA, pH 8.0 | BenchMark ULTRA |
| GPC3 | 1G12, mouse monoclonal | Roche Diagnostics | 05973864001 | Yes | According to the manufacturer’s protocol | BenchMark ULTRA |

Note: AFP, alpha-fetoprotein; ER, estrogen receptor; GPC3, glypican-3; EDTA, ethylenediaminetetraacetic acid.
