# Supplementary material for: Case Report: High-grade endometrial adenocarcinoma with somatically derived yolk sac tumor differentiation
Source: Front Oncol. 2026 Sep 3;16:1904556. doi: 10.3389/fonc.2026.1904556 (PMC13581843; doi:10.3389/fonc.2026.1904556)
Supplement: Supplementary file 2 [file Table2.docx]

| Supplementary Table 2. Summary of previously reported uterine or endometrial tumors with SDYST. | | | | | | |
| --- | --- | --- | --- | --- | --- | --- |
| Reference | Study type | Relevant cases | Main histology | Diagnostic evidence of YST differentiation | Molecular findings | Outcome relevance |
| Fadare et al., 2019 | Case series / expanded appraisal | 9 endometrial tumors classified as YST or with YST component; 3 true YST phenotypes identified in archival endometrial carcinoma/carcinosarcoma review | Endometrial tumors / carcinoma or carcinosarcoma | YST-like morphology; SALL4 and GPC3 positive; AFP variably positive | p53 aberrant in a subset | Highlights diagnostic pitfalls and need for combined morphology and IHC |
| Mills et al., 2024 | Consecutive endometrial malignancy cohort | 3 true SDYST cases among 300 endometrial malignancies | Originally diagnosed as serous, high-grade NOS, or dedifferentiated carcinoma | YST morphology plus AFP/SALL4/GPC3 expression | Included MMR status; MMR-intact tumors showed more aggressive behavior | Shows SDYST is rare but clinically important |
| Skala et al., 2020 | Molecular study | Include uterine/endometrial cases only | Mixed epithelial and germ cell features | Germ cell-like component in association with somatic carcinoma | Shared mutations support somatic derivation | Supports pathogenesis of SDYST |
| Li et al., 2026 | Clinicopathologic and molecular series | 23 primary endometrial carcinomas with SDYST | Endometrioid carcinoma, carcinosarcoma, serous carcinoma, clear cell carcinoma | Glandular, papillary, solid, endodermal sinus, hepatoid, or reticular YST patterns | Mostly p53-abnormal; no POLE-ultramutated cases reported | Shows aggressive behavior and poor outcome |
